# Bilateral Olecranon Tophaceous Gout Bursitis

**DOI:** 10.1155/2017/3514796

**Published:** 2017-02-23

**Authors:** Güzelali Özdemir, Alper Deveci, Kemal Andıç, Niyazi Erdem Yaşar

**Affiliations:** Orthopedics and Traumatology Clinic, Ankara Numune Research and Training Hospital, Ankara, Turkey

## Abstract

In this case, we present a patient with the diagnosis of bilateral olecranon tophaceous gout. After the surgical treatment, there was no limitation of range of motion or wound problem at 6th month control.

## 1. Introduction

Gout is a metabolic disease of monosodium urate crystal deposition and the peak age incidence occurs at 30–50 years [1]. Chronic tophaceous gout occurs in patients who have had poorly controlled gout for a prolonged period of time [2]. Tophaceous gout occurs in less than 10% of gout patients [1].

Tophaceous disease is usually preventable given the availability of effective urate lowering medication therapies (ULT). Despite the medical therapy, there remains a subset of patients who develop significant complications of tophi including infection, ulceration, and entrapment neuropathy. Tophi in close proximity to joints can cause joint instability, severely limited range of motion, and significant functional impairment [3].

The predilections of this disease process are the feet, knees, hands, the cartilages of the ear, around the olecranon bursa, and the nose [1]. On the other hand, there can be unusual presentations associated with chronic gout. Presentation with bilateral olecranon chronic tophaceous gout was reported very rarely [4].

The aim of this study is presenting a case of bilateral tophaceous gout over the olecranon.

## 2. Case Report

59-year-old man attended our clinic with bilateral masses. The patient reported having a history of gout disease and use of colchicine. He was ex-smoker and with history of hypertension.

Clinically solitary, nonmobile, 4 cm*∗*3 cm sized, nontender, firm masses were seen over the posterior aspect of both elbows. Swellings were not painful but associated with 50 restrictions of elbow flexion movement. There were no signs of inflammation detected. He has no history of trauma, fever, discharging sinus, other joints involvement. Apart from these, the patient was completely clinically normal.

**Figure 1:**
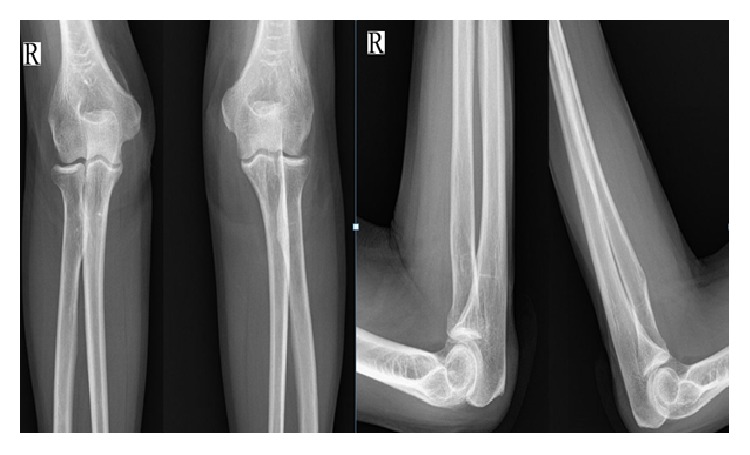
Images of X-ray, right and left elbow: The direct preoperation radiographies showed no significant changes or calcification (9/5/16).

The patient had serum uric acid elevated at 11 mg/dl (elevated, normal 3,5–7,2 mg/dl), normal serum urea of 31 mg/dl (normal 17–43 mg/dl), normal serum creatinine of 1,14 mg/dl (normal 0,81–1,44 mg/dl), normal calcium of 9,7 mg/dl (normal 8,5–10,5), normal erythrocyte sedimentation rate of 15 mm/h (normal 0–20 mm/h), and C-reactive protein of 3 mg/L (normal 0–5 mg/L). The direct radiographies did not show any significant changes or calcification (Figure 1).

Magnetic Resonance Imaging (MRI) of the right elbow reported the following: intra-articular minimal effusion, at the site of the posterior bursa of olecranon in T2a sections and hyper-T1a sections, hypoisointense, approximately 44*∗*21*∗*32 mm sized, complicated tissue, although IV contrast MRI studies obtained nothing but minimal changes.

**Figure 2:**
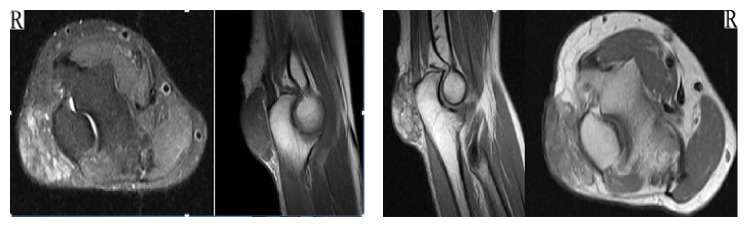
Preoperation right elbow MRI images, T1a and T2a sections, at site of the posterior bursa of 44 × 21 × 32 mm sized complicated tissue. No destruction of bone (9/5/16).

MRI study of the left elbow reported the following: intra-articular minimal effusion, minimal degeneration changes, in T2a and T1a sections, lobule projected hypointense approximately 46*∗*22*∗*34 mm sized tissue, and complicated bursitis (Figure 2).

There was a surgical indication due to the aesthetic concern of the patient. After tests, he consulted an anesthesiologist and then ASA-2 open bursa excisions were bilaterally applied under general anesthesia.

## 3. Surgical Treatment

The procedure, alternatives, risks, and limitations in this individual case have been very carefully discussed with the patient. All questions have been thoroughly answered, and the informed consent was signed.

**Figure 3:**
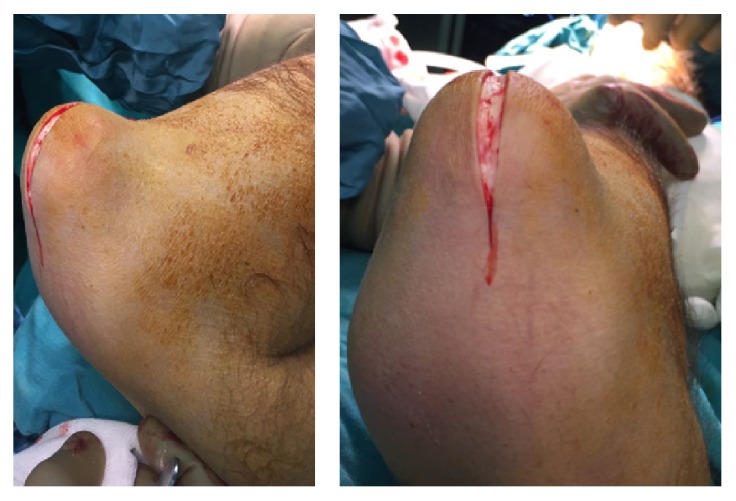
Posterior longitudinal approach of the left elbow.

The patient was placed in supine position on the operating table. A general anesthetic was administered, and extremities was prepped and draped in the usual sterile manner. The upper limbs were wrapped with Esmarch bandages and a previously placed well-padded pneumatic tourniquet was inflated. Longitudinal 7 cm incision was used (Figure 3).

**Figure 4:**
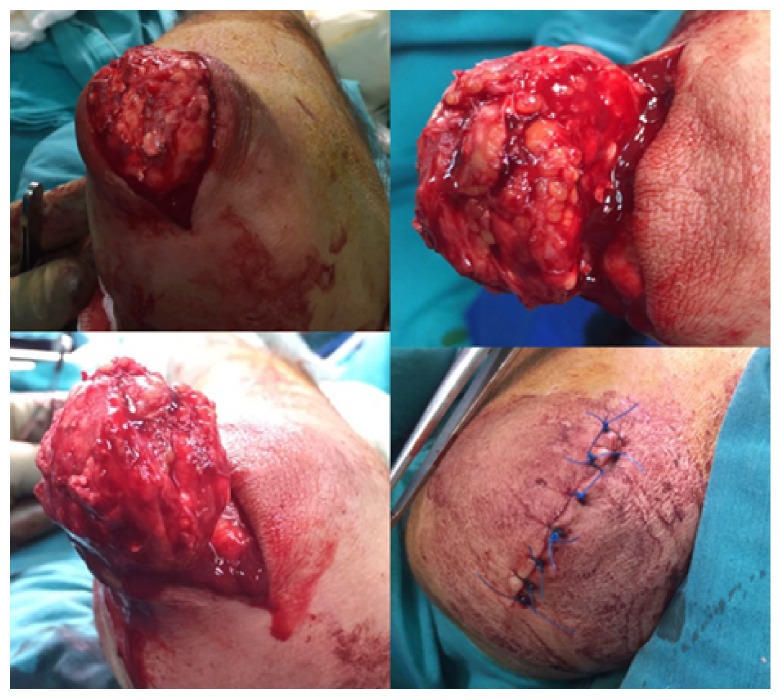
The mass was enucleated and dissected. Preoperation photographs of the left elbow mass; before removal and after closure of the skin (16/5/16).

After dissection of the skin, we encountered calcified cystic tissues. The mass separated from tissues by blunt dissection. During the surgery, we did not see any pedicle or communication with the elbow joint. The mass was enucleated from wound and removed (Figure 4).

**Figure 5:**
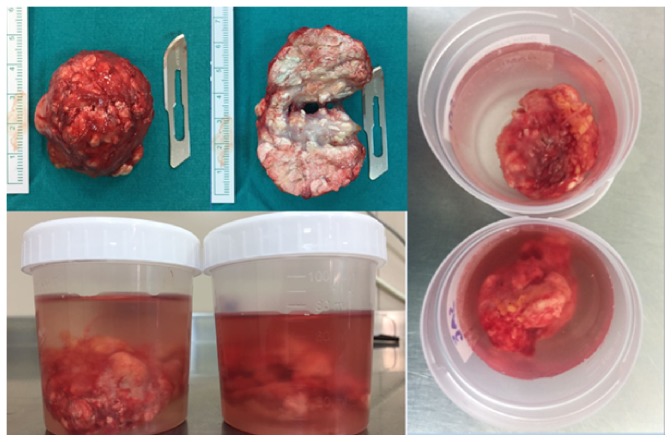
Two pieces of the specimens about 4.5 cm each, prepared and sent for pathologic examination.

The wounds were irrigated with saline. The tourniquet was deflated. Homeostasis of small bleeding points stopped using bipolar cautery. The skin wound was closed in layers with buried deep dermal 2-0 vicryl and 2-0 nylon. The wound was dressed. And the same procedure executed for the other extremity. The patient tolerated the procedure well and was awakened in the operating room and transported to the recovery room in stable condition. Specimens were sent for histopathological examination (Figure 5).

## 4. Pathology

Right elbow pathology findings were as follows: 5,2 *∗* 4 *∗* 3 cm sized white-yellow-brown colored, thin light fibrous capsule like covered tissue; dirty white colored tissues were seen in the sections. Left elbow pathology findings were as follows: 5 *∗* 4,5 *∗* 3 cm sized dirty white-yellow-brown colored thin fibrous capsule like covered tissue; in sections white and yellow nodules were seen. And the results were reported as compatible with tophaceous gout.

## 5. Postoperative Care

**Figure 6:**
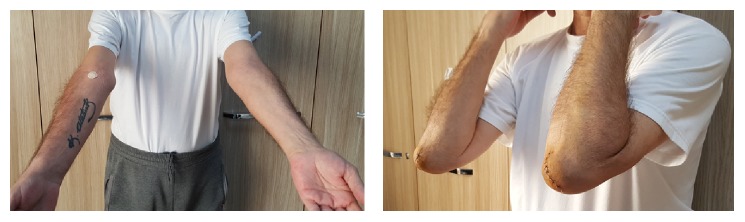
Postoperative 17th day, patient reached full joint range of motion (2/6/16).

On the next day following surgery, passive and active range of motion exercises started aggressively and the patient was discharged. The patient was referred to an internal medicine specialist for medical treatment of gout disease. Every other day, wound care was done. The patient did not experience any complications. At the 17th day, sutures were removed. He returned to work at the 20th day. There was no limitation of range of motion or wound problem at the 1st, 3rd, and 6th month's controls (Figure 6).

## 6. Discussion

Gout is a systemic-metabolic disease with various symptoms. Clinic symptoms may appear in patients who had untreated asymptomatic hyperuricemia for a long time. Disease can develop recurring arthritis attacks following asymptomatic intercritical episodes in the end chronic arthritis and tophaceous may develop. Using the patient's history, the finding from physical examination, the laboratory findings, and radiologic features found on plain radiographs, CT, or MRI are all helpful in making the proper diagnosis of chronic tophaceous gout.

Gout may cause bursitis in general, and the olecranon bursa is one of the most affected ones. The olecranon bursa is very commonly involved in tophaceous gout because of the tendency of monosodium urate crystals to deposit in superficial structures with low temperatures [5]. But however bilateral olecranon tophaceous gout is very rare [4]. Chronic gout often causes boney erosions, which lead to debilitating joint damage. Characteristic features of joint damage boney erosions are new bone formation, tophi within tendons, and focal cartilage loss with eventual joint destruction [6]. MRI is not routinely used for evaluation of tophaceous gout. However, gout may present clinically in an atypical, unusual, or confusing manner. A gouty tophus occasionally mimics an infectious or neoplastic process and MRI must be obtained under these circumstances [7]. But in this case there were no signs of infection or neoplastic processes and any joint destruction or bone erosions had shown.

In this case, the effects of untreated long-term gout were reduced mobility and aesthetic problems. Tophaceous gout can be presented at the various joints of the body especially knee, 1st metatarsophalangeal, and elbow joints. But unusual presentations may occur. Despite the early recognition and medication, chronic problems could be happen. But in cases like we present and well established in joints, surgical excision could be the only solution to solve minimally decreased range of motion and aesthetic problems.

## 7. Conclusion

Tophaceous gout may be presented with unusual clinical manifestations. Bilateral tophaceous gout over the olecranon should be considered in differential rare diagnosis of the olecranon bursitis. Metabolic problems should be considered a priority with the patients who have bilateral joints problems.
